# Development of recombinant inbred lines and QTL analysis of plant height and fruit shape-related traits in *Cucurbita pepo* L.

**DOI:** 10.1007/s11032-025-01592-y

**Published:** 2025-09-09

**Authors:** Ying Duan, Kailiang Bo, Qin Shu, Meng Zhang, Yuzi Shi, Yiqun Weng, Changlin Wang

**Affiliations:** 1 State Key Laboratory of Vegetable Biobreeding, Institute of Vegetables and Flowers, Chinese Academy of Agricultural Sciences, Beijing, 100081 China; 2 Plant Science and Agroecosystem Department, USDA-ARS Vegetable Crops Research Unit, University of Wisconsin, Madison, WI 53706 USA; 3 Beijing Key Laboratory of Growth and Developmental Regulation for Protected Vegetable Crops, College of Horticulture, China Agricultural University, Beijing, 100193 China

**Keywords:** *Cucurbita pepo*, Recombinant inbred lines, High-density genetic map, Plant height, Fruit shape

## Abstract

Zucchini (*Cucurbita pepo* subsp. *pepo*) stands as an economically vital crop in China. In zucchini breeding, plant architectural patterns and fruit morphological characteristics serve as pivotal traits. In this study, we employed quantitative trait locus (QTL) analysis using recombinant inbred lines (RILs) derived from two distinct inbred lines, JinGL (subsp. *ovifera*) and HM-S2 (subsp. *pepo*), in conjunction with a high-density genetic map. Our investigation focused on ten QTLs associated with six horticulturally significant traits, including hypocotyl length (HL), plant height (PH), and four fruit-related traits: fruit length (FL), fruit diameter (FD), fruit shape index (FSI), and fruit weight (FW). The QTLs governing HL and PH were mapped to Chr03/LG10 and named *qhl3.1* and *qph3.1*, respectively. The candidate gene *Cp4.1LG10g05910*/*CpDw* for *qph3.1* was successfully identified. Additionally, three novel QTLs related to fruit size and shape were discovered. Among them, *qfsi8.1/qfl8.1*, demarcated by Marker238258 and Marker240069 on Chromosome 08/Linkage group 17 (Chr08/LG17), is a new major QTL regulating the fruit shape of zucchini. Through genomic insertion-deletion (InDel) and qRT-PCR analyses, we predicted genes within the *qfsi8.1/qfl8.1* candidate interval, uncovering *Cp4.1LG17g02030/CpIAA12* and *Cp4.1LG17g02010/CpCalB* as potential candidate genes. We developed molecular markers tightly linked to *qph3.1* and *qfl8.1* and validated them in 171 and 224 *Cucurbita pepo* germplasms, achieving accuracy rates of 96% and 100%, respectively. This study deepens our understanding of the genetic basis of key traits and provides valuable references for molecular breeding in *Cucurbita pepo*.

**Supplementary Information:**

The online version contains supplementary material available at 10.1007/s11032-025-01592-y.

## Introduction

*Cucurbita pepo* is a globally cultivated crop of economic importance (Sanjur et al. 2002; Paris 2016). It exhibits remarkable diversity in fruit characteristics compared to many other cucurbit crops (Gong et al. 2012; Xanthopoulou et al. 2019). Based on distinctive fruit phenotypic characteristics, at least eight cultivation groups of *Cucurbita pepo* have been recognized, including zucchini, acorn, and scallop (Ferriol et al. 2003; Wang et al. 2021).

In *Cucurbita pepo*, several genetic maps employing diverse molecular markers and segregating populations have been constructed to identify quantitative trait loci (QTL) associated with horticultural traits. For example, Gong et al. (2008a, b) developed an SSR-based genetic map and detected four SSR markers linked with the *hull-less seed* locus (*h*) on LGp9, and a major-effect QTL for the *bush* locus (*B*). We developed a linkage map with 437 EST-SSR marker loci, and identified QTL for vine length and internode length variation; we also predicted that the gene *Cp4.1LG10g05910* encodes a GA2 oxidase as the candidate for the vine length major-effect QTL (Xiang et al. 2018). This result was subsequently verified through bulked segregant analysis (BSA) and fine genetic mapping using InDels or KASP markers (Ding et al. 2021). Esteras et al. (2012) conducted QTL analysis using an F_2_ population derived from the cross between zucchini and scallop (referred to as ‘Fei Die-gua’ in Chinese). This genetic linkage map contained 315 marker loci in 22 linkage groups (LG), and 48 QTLs for multiple morphological traits were identified, including leaf blade, silvered leaves, flowering, fruit shape, and fruit color. Subsequently, Montero-Pau et al. (2017) used the same parental lines and developed a genetic map with RILs from the same cross, which contained 7718 marker loci in 15 linkage groups. This immortal RIL population enabled multiple-environment and replicated trait analyses. These examples in *Cucurbita pepo* highlight the power of developing high-density genetic maps using high-throughput sequencing technologies as well as immortal segregating populations.

Although *Cucurbita pepo* exhibits rich diversity in fruit types and flesh colors (Obrero et al. 2013; Xu et al. 2021; Hernandez et al. 2023), the predominant cultivated type in China and East Asia is *Cucurbita pepo* subsp. *pepo* (zucchini, referred to as ‘Xi Hu-lu’ in Chinese). Zucchini breeding prioritizes key traits including compact plant architecture, uniform fruit shape/color, and moderate fruit size. However, zucchini breeding is facing long-standing challenges: a narrow genetic background in germplasm, limited diversity in cultivated varieties, and significant reliance on imported varieties. Moreover, the lack of high-quality genetic maps and functional molecular markers has constrained the advancement of molecular-assisted breeding in zucchini.

To address these limitations, two inbred lines were selected as parental lines. HM-S2, a typical pepo-type zucchini, represents a globally common cultivar of *Cucurbita pepo*; in contrast, JinGL (*Cucurbita pepo* subsp. *ovifera*), known as ‘Ju Gua’ (acorn type), is widely cultivated in East Asia and southern China. Agronomic evaluations (Wyatt et al. 2015, 2016; Bo et al. 2022) have documented that the acorn type exhibits ornamental value, palatability, distinct leaf morphology, high fruit set efficiency, and superior fruit quality. The utilization of these unique traits can effectively enhance the innovation potential of zucchini breeding by introducing novel genetic diversity.

In this study, a recombinant inbred line (RIL) population was constructed from a cross between two inbred lines representing the zucchini and acorn cultivation groups. Using specific-locus amplified fragment sequencing (SLAF-seq) (Baird et al. 2008; Elshire et al. 2011; Sun et al. 2013), we developed a high-density genetic linkage map. Through multi-year, multi-environment evaluations, phenotypic variation was evaluated for key horticultural traits. Candidate genes underlying major QTLs associated with plant height and fruit shape were identified, and tightly linked molecular markers were developed to facilitate marker-assisted breeding. These findings provide theoretical insights into the genetic analysis and utilization of horticultural traits in *Cucurbita pepo*.

## Materials and methods

### Plant materials and phenotypic data collection

An RIL population consisting of 171 lines was generated through single-seed descent (SSD) from the cross between two inbred lines JinGL (P_1_) and HM-S2 (P_2_). Phenotypic data for six traits including hypocotyl length (HL), plant height (PH), and mature fruit-related traits (FL/FD/FW) in the RIL population were collected in six years across four locations. The fruit shape index (FSI) was calculated as FL/FD. The details of all experiments are presented in Table 1.

**Table 1:** Experiment design of different environments in five years for QTL analysis

Experiments	Seasons	Locations	Traits*
2016 S	2016 Spring	Langfang, Hebei, China	PH
2016 F	2016 Fall	Langfang, Hebei, China	FL, FD, FSI
2017 S	2017 Spring	Langfang, Hebei, China	FL, FD, FSI
2018 S	2018 Spring	Langfang, Hebei, China	PH
2018 F	2018 Fall	Shunyi, Beijing, China	FL, FD, FSI, FW
2019 S	2019 Spring	Shunyi, Beijing, China	PH, FL, FD, FSI, FW
2021 S	2021 Spring	Linxi, Hebei, China	PH, FL, FD, FSI, FW
2021March	2021 Spring	Haidian, Beijing, China	HL
2021April	2021 Spring	Haidian, Beijing, China	HL
2021May	2021 Spring	Haidian, Beijing, China	HL

* PH plant height, *FL* fruit length, *FD* fruit diameter, *FSI* fruit shape index, *FW* fruit weight, *HL* hypocotyl length

All plants were grown in the greenhouses of the Experimental Stations. Seeds were sown in 32-hole trays filled with organic substrate and perlite (3:1, v/v). After 14 days, the seedlings were transplanted into soil. In each trial, there were 12 plants from each parental line and 6 plants of each RIL per row/plot with 0.5 m spacing between plants and 0.8 m between rows.

For HL, three experiments were performed in March, April, and May of 2021. In each experiment, HL of 10-day-old seedlings grown in 32-hole trays was measured using a ruler. Three biological replicates were performed for each RIL line, with 10 plants measured per replicate. For PH data collection, the length of the main stem from the tip to the attachment point of soil was measured. FL and FD were measured with a ruler, and FW was measured with a scale, for mature fruits at 45 days after pollination (DAP). FSI was calculated from FL/FD. In each trial, the PH, FD, and FW data were the means from three plants (out of a total of 6) for each RIL.

### Resequencing, SLAF-seq and high-density linkage map construction with RILs

Genomic DNA was extracted from young leaves of two parental lines and 171 RILs using the CTAB (cetyl trimethyl ammonium bromide) method (Murray and Thompson 1980). The integrity of DNA samples was assessed by 1% agarose gel electrophoresis, and the DNA was quantified with NanoDrop 2000 (Thermo Scientific, USA). The two parental lines underwent high-throughput re-sequencing, while SLAF-seq was performed on the 171 RILs.

The general procedure to develop SLAF markers followed Sun et al. (2013), which was conducted by Biomarker Technologies Company (Beijing, China) as a commercial service. During the assessment of the reference genome quality, we found that the genome assembly of *Cucurbita pepo* (Montero-Pau et al. 2018) was suboptimal. Given the high level of collinearity among the three major cultivated species in the *Cucurbita* genus, we utilized *Cucurbita maxima* cv. Rimu as the reference genome (Sun et al. 2017), which has a higher-quality genome assembly. Comparative analyses suggested a high degree of collinearity between the two genomes (Zhang et al. 2015; Xiang et al. 2018; Zeng et al. 2024). SLAF-seq library preparation and sequencing followed Sun et al. (2013). The libraries were generated using double digestion with two restriction enzymes, *Hae*III and *Hpy*166II (New England Biolabs, NEB, USA), which were predicted to generate about 168 000 SLAFs in the *Cucurbita maxima* genome.

After filtering the raw reads, clean reads were mapped to the * Cucurbita maxima* genome using the SOAP (Li et al. 2008). Single nucleotide polymorphisms (SNPs) within each SLAF locus were detected using GATK with a series of strict filtering rules following the guidelines provided at https://www.broadinstitute.org/gatk/guide/best-practices.php. Polymorphic SLAFs were selected with the following criteria: (1) biallelic and reliable with > 10× coverage of sequencing depth in the parents; (2) homozygous in the RIL carrying the allele of either parent; (3) missing rate among 171 RILs < 30%; (4) no more than 3 SNPs in each SLAF locus; (5) showed no severe segregation distortion (*P* < 0.01 in *Chi-square test*). Finally, 9117 polymorphic SLAF markers that passed these filtering criteria were retained for linkage analysis in HighMap, with a modified logarithm of odds (MLOD) score > 5.0 as the threshold.

To improve the accuracy of linkage grouping, an error correction strategy known as SMOOTH was implemented, which considers the parental contributions of genotypes and helps correct errors in the marker order and genetic distances (van Os et al. 2005). The K-nearest neighbor algorithm was utilized to impute missing genotypes (Huang et al. 2011). This algorithm estimates the missing genotypes based on the genotypes of nearby markers and fills in the genetic map with the most likely value for the missing data. Map distances between markers were estimated using the Kosambi mapping function (Kosambi 1943), which uses recombination frequencies to calculate genetic distances between markers on the linkage map.

### QTL analysis and candidate gene prediction in target QTL regions

QTL analysis was conducted using *R/qtl* 1.70 package (http://www.rqtl.org/) with the composite interval mapping (CIM) method (Jansen 1993; Broman et al. 2003). To determine the genome-wide LOD threshold for identifying significant QTLs, 1000 permutations were performed and a significance level of *P* = 0.05 was used for determining the threshold. For each detected QTL, a 1.5-LOD-support interval was calculated, defined by left and right markers following Dupuis and Siegmund (1999). The PVE values were calculated using the *fitqtl()* function from the *R/qtl* package (Broman et al. 2003). For each identified QTL, the PVE was derived from the proportion of variance explained by the QTL model relative to the null model, with the formula: PVE=[(1-*RSS_*_*QTL*_/*RSS_*_*null*_)] × 100%. Herein, *RSS_*_*QTL*_ represents the residual sum of squares of the model including the QTL, while *RSS_*_*null*_ denotes the residual sum of squares of the null model (with only the intercept term). This method is consistent with the widely adopted approaches in QTL mapping studies.

The sequences of candidate genes within the identified QTLs were analyzed using the *Cucurbita pepo* (http://cucurbitgenomics.org/ftp/genome/Cucurbita_pepo/) and *Cucurbita maxima* (http://cucurbitgenomics.org/ftp/genome/Cucurbita_maxima/v1.1/) reference genomes.

### qRT-PCR analysis

Four inbred lines of *Cucurbita pepo* with distinct fruit morphologies were used as experimental materials to investigate the expression levels of candidate genes. The fruits (ovaries) of female flowers on the day of pollination were rapidly frozen in liquid nitrogen and then stored at −80 °C. The total RNA was extracted using the MiniBEST Universal RNA Extraction Kit (TaKaRa, 9769). First-strand cDNA was synthesized using the PrimeScript™ RT reagent Kit with gDNA Eraser (Perfect Real Time) (TaKaRa, RR047A). The OligoAnalyzer tool (https://sg.idtdna.com/calc/analyzer) was employed to design primers for qRT-PCR for six candidate genes (Table S7). The reaction system had a total volume of 10 µL, consisting of 5 µL of 2× ChamQ Universal SYBR qPCR Master Mix (Vazyme, Q711), 1 µL of cDNA, 0.5 µL of each forward and reverse primer, and 3 µL of ddH₂O. The reaction program was as follows: initial denaturation at 95 °C for 3 min; followed by 39 cycles of denaturation at 95 °C for 15 s, annealing at 60 °C for 30 s, with fluorescence signal collection at the third step of each cycle; then a melting curve step starting at 65 °C, with the temperature increased by 0.5 °C every 5 s until reaching 95 °C. Three biological replicates and three technical replicates were performed for each sample. The obtained data were analyzed using the 2⁻^ΔΔCt^ method (Livak and Schmittgen 2001).

### Validation of molecular markers

We developed InDel markers based on these polymorphisms to analyze genotype-phenotype association in 171 (for HL) and 224 (for FL) *Cucurbita pepo* accessions. Locus-specific PCR primers were designed using the OligoAnalyzer tool (https://sg.idtdna.com/calc/analyzer) and synthesized by Sangon Biotech (Shanghai, China). Genomic DNA of the accessions served as template for PCR reactions conducted in 20 µL volumes, containing 2 µL of DNA template, 2 µL of each primer (20 µM), 6 µL of ddH_2_O, and 10 µL of 2× PCR reaction mix (Vazyme, Nanjing, China). The PCR program included an initial denaturation at 95 °C for 2 min, followed by 35 cycles of denaturation (95 °C, 20 s), annealing (56–58 °C, 20 s), and extension (72 °C, 30 s), with a final extension step at 72 °C for 10 min after the last cycle. PCR products were resolved by 6.5% denaturing polyacrylamide gel electrophoresis (PAGE) and visualized by silver staining.

## Results

### Development of a high-density genetic map with SLAF-seq

High-throughput Illumina sequencing of the two parental lines (JinGL and HM-S2) generated 25.0 and 29.1 million high-quality reads, with total sequence lengths of 7.5 Gbp and 8.7 Gbp, respectively. This resulted in sequencing depths of 31.1× for JinGL and 26.7× for HM-S2, relative to the reference genome. After initial filtering, 91.2% of clean reads (mean Q30 = 94.52%) were mapped to the *Cucurbita maxima* cv. Rimu reference genome (Table S1). Following a series of polymorphism screening steps (see Methods for criteria), 9117 SLAF markers were selected for linkage analysis.

**Fig. 1:**
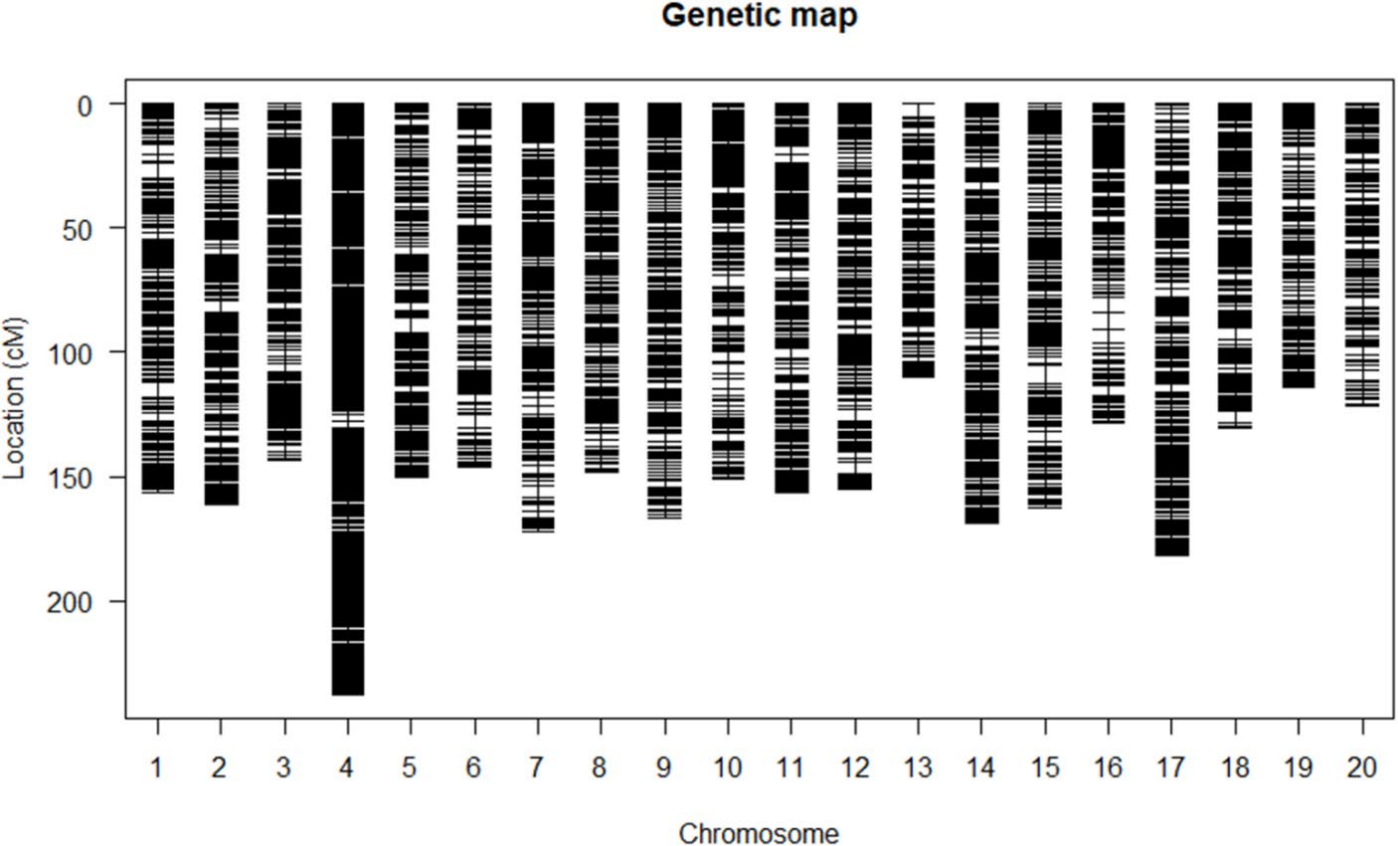
Graphic distribution of 9117 SLAF markers on a high-density genetic map constructed using 171 RILs derived from a cross between inbred lines JinGL and HM-S2. Black lines indicate the linear arrangement of SLAF markers on each linkage group. Each linkage group corresponds to one of the 20 chromosomes of *Cucurbita pepo*

All 9117 SLAF markers were assigned to 20 linkage groups (LGs), which correspond to the 20 chromosomes of the *Cucurbita maxima* reference genome. A schematic of the linkage map is shown in Fig. 1. Alignment of SLAF markers to the *Cucurbita maxima* cv. Rimu genome revealed strong collinearity in marker order between the genetic map and physical assembly for each chromosome (Fig. S1). Spearman’s correlation coefficients between physical and genetic orders for each LG ranged from 0.964 to 1.000, indicating nearly complete genome coverage and high quality of the high-density genetic map. Key statistics of the genetic map are summarized in Table 2. The total genetic length of the map was 3062.6 cM, with a mean inter-marker distance of 0.34 cM. The largest gaps were observed on Chr15 (6.98 cM), with a mean LG length of 153.1 cM. The shortest and longest LGs were Chr13 (109.9 cM; 236 marker loci) and Chr4 (237.6 cM; 921 marker loci), respectively.

**Table 2:** Main statistics of high-density genetic map

Chromosome	Loci	Map Length (cM)	Mean Interval (cM)	Max. Gap (cM)	Gap < 5 cM (%)
Chr01	638	156.24	0.25	6.24	99.69
Chr02	581	161.12	0.28	4.62	100.00
Chr03	440	143.46	0.33	3.34	100.00
Chr04	921	237.62	0.26	2.63	100.00
Chr05	412	150.43	0.37	6.04	99.76
Chr06	409	146.39	0.36	5.46	99.75
Chr07	414	171.88	0.42	4.75	100.00
Chr08	400	148.26	0.37	2.61	100.00
Chr09	392	166.88	0.43	3.17	100.00
Chr10	386	151.19	0.39	5.06	99.74
Chr11	542	156.34	0.29	4.49	100.00
Chr12	386	155.10	0.40	4.71	100.00
Chr13	236	109.92	0.47	5.48	99.75
Chr14	665	168.92	0.25	3.17	100.00
Chr15	366	162.63	0.45	6.98	99.73
Chr16	330	128.13	0.39	6.75	99.09
Chr17	456	181.66	0.40	3.64	100.00
Chr18	381	130.70	0.34	5.09	99.74
Chr19	355	114.07	0.32	3.38	100.00
Chr20	407	121.63	0.30	3.86	100.00
Total	9117	3062.57	0.34		99.85

### Phenotypic variation and QTL analysis for hypocotyl length and plant height

**Fig. 2:**
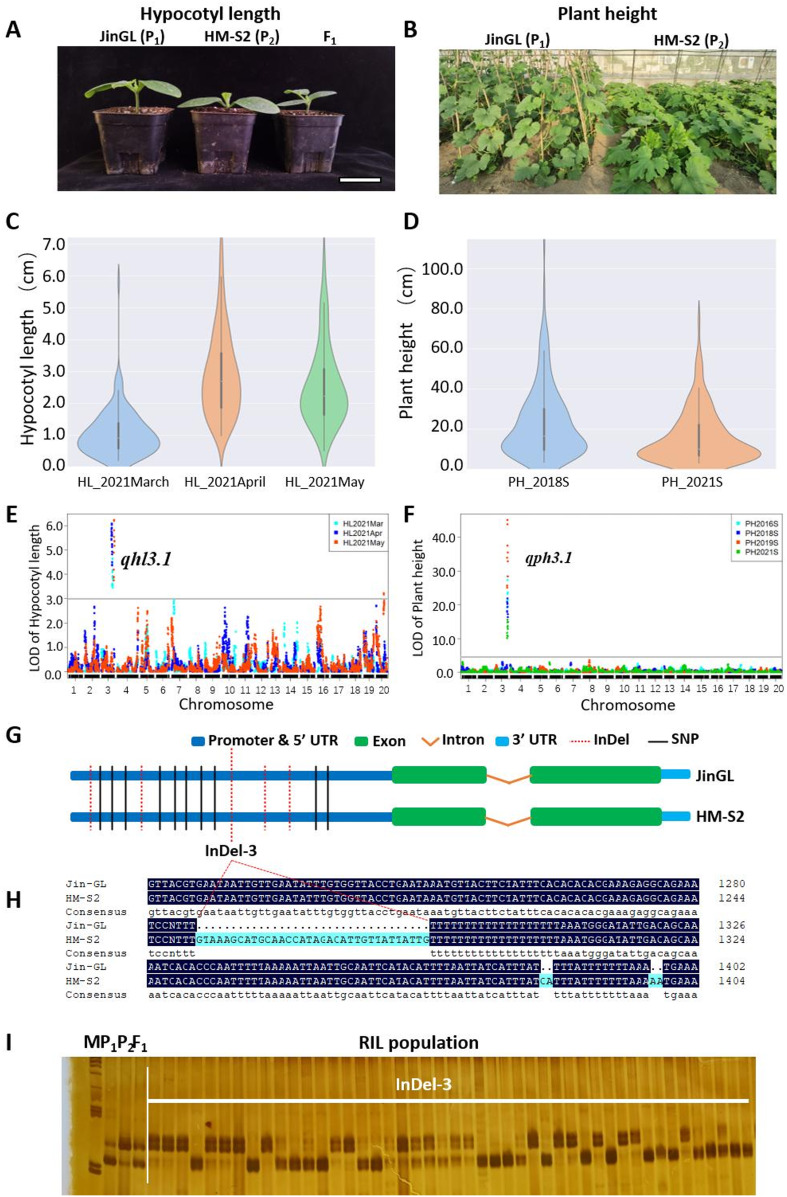
Identification of candidate genes underlying major-effect QTL for hypocotyl length (HL) and plant height (PH) variation in the RIL population. (**A)** Hypocotyl length of 10-day-old seedlings in JinGL, HM-S2 and F_1_. Scale bar = 5 cm. (**B**) Plant height of adult JinGL and HM-S2 plants in a greenhouse. **(C**,** D)** Violin plots showing phenotypic variation in HL (C) and PH (D) among RILs in different environments. (**E**,** F**) LOD profiles from QTL analysis for HL (E) and PH (F) using data from different environments. A single major-effect QTL was consistently detected for HL (*qhl3.1*), and PH (*qph3.1*), which colocalized on Chr03/LG10. (**G**) *Cp4.1LG10g05910*/*CpDw* is a candidate gene underlying *qph3.1*. (**H**) Sequence alignment of *Cp4.1LG10g05910/CpDw* between JinGL and HM-S2, highlighting InDels and SNPs in the promoter region. (**I**) Validation of the InDel-3 marker for PH, showing co-segregation with phenotypes in the RIL population

Hypocotyl length (HL) was measured in 10-day-old seedlings of 171 RILs across three experiments conducted in March, April and May 2021. Plant height (PH) data were recorded 30 days after transplanting to the field. JinGL consistently exhibited longer HL than HM-S2, with 1.75-, 1.77-, and 1.75-fold increases in the three experiments, respectively (Fig. 2A; Table 3). Similarly, the adult plant height of JinGL exceeded that of HM-S2 by 5.28-fold in the 2018 growing season (2018 S) and 8.16-fold in the 2021 season (2021 S). The F_1_ progeny showed a 4.26-fold increase in plant height compared to HM-S2 in 2018 S and was 3.76-fold taller in 2021 S (Fig. 2B; Table 3). In all seasons, both HL and PH exhibited continuous phenotypic distributions across the RIL population (Fig. 2C-D).

**Table 3:** Statistical data of agronomic traits in parental lines and RILs

Trait	Environments	Parental Lines		F_1_			RILs		
		P_1_ JinGL	P_2_ HM-S2		Min	Max	Mean	Std	CV
HL/cm	2021March	1.4 ± 0.3	0.8 ± 0.2	1.0 ± 0.3	0.2	5.8	1.1	0.7	0.68
	2021April	1.6 ± 0.4	0.9 ± 0.2	1.1 ± 0.4	1.1	6.8	1.6	1.3	0.46
	2021May	2.8 ± 0.7	1.6 ± 0.5	2.4 ± 0.5	0.5	6.5	2.6	1.3	0.49
PH/cm	2018 S	145.2 ± 7.8	27.5 ± 2.6	117.2 ± 0.5	3.4	114.6	23.6	18.2	0.77
	2021 S	98.8 ± 9.0	12.1 ± 1.4	45.5 ± 3.5	3.2	84.2	16.1	13.3	0.83
FL/cm	2016 F	6.8 ± 0.2	37.3 ± 0.8	26.6 ± 1.4	6.0	46.8	16.1	6.3	0.39
	2017 S	6.7 ± 0.3	37.6 ± 0.8	28.2 ± 1.1	5.3	34.5	15.0	5.9	0.39
	2018 F	6.3 ± 0.1	38.9 ± 0.4	16.5 ± 0.2	5.5	47.8	17.7	6.8	0.39
	2019 S	7.6 ± 0.7	33.2 ± 3.2	26.7 ± 3.3	4.8	45.5	17.5	6.9	0.40
	2021 S	7.0 ± 0.3	36.7 ± 0.8	26.6 ± 1.4	3.9	37.5	15.9	6.6	0.41
FD/cm	2016 F	8.7 ± 0.3	10.5 ± 0.1	10.3 ± 0.2	4.7	14.9	9.2	1.8	0.19
	2017 S	8.9 ± 0.2	10.8 ± 0.5	11.0 ± 0.3	3.7	14.7	8.4	1.9	0.23
	2018 F	8.4 ± 0.1	10.8 ± 0.6	10.0 ± 0.2	5.4	15.6	9.7	2.0	0.20
	2019 S	8.6 ± 0.2	8.5 ± 0.7	10.8 ± 0.3	4.9	15.6	9.5	2.4	0.26
	2021 S	9.2 ± 0.6	10.8 ± 0.7	11.1 ± 0.3	4.5	14.5	9.3	2.0	0.21
FSI	2016 F	0.8 ± 0.0	3.5 ± 0.1	2.6 ± 0.1	0.6	4.8	1.5	0.3	0.50
	2017 S	0.7 ± 0.0	3.5 ± 0.2	2.5 ± 0.1	0.6	6.3	1.5	0.4	0.52
	2018 F	0.8 ± 0.1	3.6 ± 0.2	1.7 ± 0.1	0.6	4.8	1.4	0.4	0.52
	2019 S	0.9 ± 0.2	3.9 ± 0.8	2.5 ± 0.2	0.4	5.0	1.9	0.4	0.57
	2021 S	0.8 ± 0.1	3.4 ± 0.1	2.4 ± 0.1	0.6	4.4	1.8	0.5	0.46
FW/g	2018 F	284.9 ± 38.1	2339.5 ± 93.1	1384.3 ± 96.8	74.3	2542.0	750.1	434.3	0.57
	2019 S	383.5 ± 24.4	2217.5 ± 103.3	1668.4 ± 78.4	77.9	2318.0	791.6	377.6	0.48
	2021 S	379.8 ± 15.6	2481.0 ± 97.9	1642.0 ± 69.6	80.0	2970.0	739.7	420.9	0.57

*Std* Standard Deviation, *CV* Coefficient of Variation

QTL analysis using data from different seasons consistently identified one major-effect QTL for HL (designated as *qhl3.1*) and one for PH (designated as *qph3.1*) on Chr03/LG10. *qhl3.1* explained 15.1–15.8% of PVE, whereas *qph3.1* accounted for 30.1–70.9% of PVE (Table 3). To our knowledge, no hypocotyl-length-related QTLs have been reported in *Cucurbita pepo* previously, suggesting that *qhl3.1* represents a novel QTL governing hypocotyl development in this species.

The 1.5-LOD interval for *qph3.1* spanned approximately 2.42 cM, corresponding to a 219.7-kb physical region between 522 065 and 741 775 on Chr03/LG10. This interval contained 43 genes, including the candidate gene *Cp4.1LG10g05910* (*also designated CpDw*), which encodes a *GA2ox2* enzyme involved in vine length regulation by deactivating bioactive gibberellins (Ding et al. 2021). The QTLs *qph3.1* and *qhl3.1* were colocalized on Chr03/LG10 (Fig. 2E-F; Table 3), with their physical positions either closely adjacent or overlapping (Fig. 2E-F).

Sanger sequencing of the *Cp4.1LG10g05910/CpDw* promoter region (2 000 bp upstream of the start codon) and coding region in parental lines JinGL and HM-S2 revealed five InDels and 10 SNPs, with no nucleotide differences detected in the coding region between the two parental lines (Fig. 2G). A specific InDel polymorphism at −751 bp upstream of the start codon was developed into the InDel marker InDel-3, which was used to genotype the RIL population (Fig. 2H). Genotyping results showed that the two alleles of PH-InDel-3 marker co-segregated with plant height phenotypes in all 171 RILs (Fig. 2I), supporting that *Cp4.1LG10g05910/CpDw* also functions as a plausible candidate gene for the QTL *qph3.1*. Whether *Cp4.1LG10g05910/CpDw* also functions as the candidate gene for hypocotyl length variation requires further validation. These findings highlight the utility of high-density genetic mapping for rapid and high-resolution QTL detection.

### QTL analysis of fruit size/shape-related traits

**Fig. 3:**
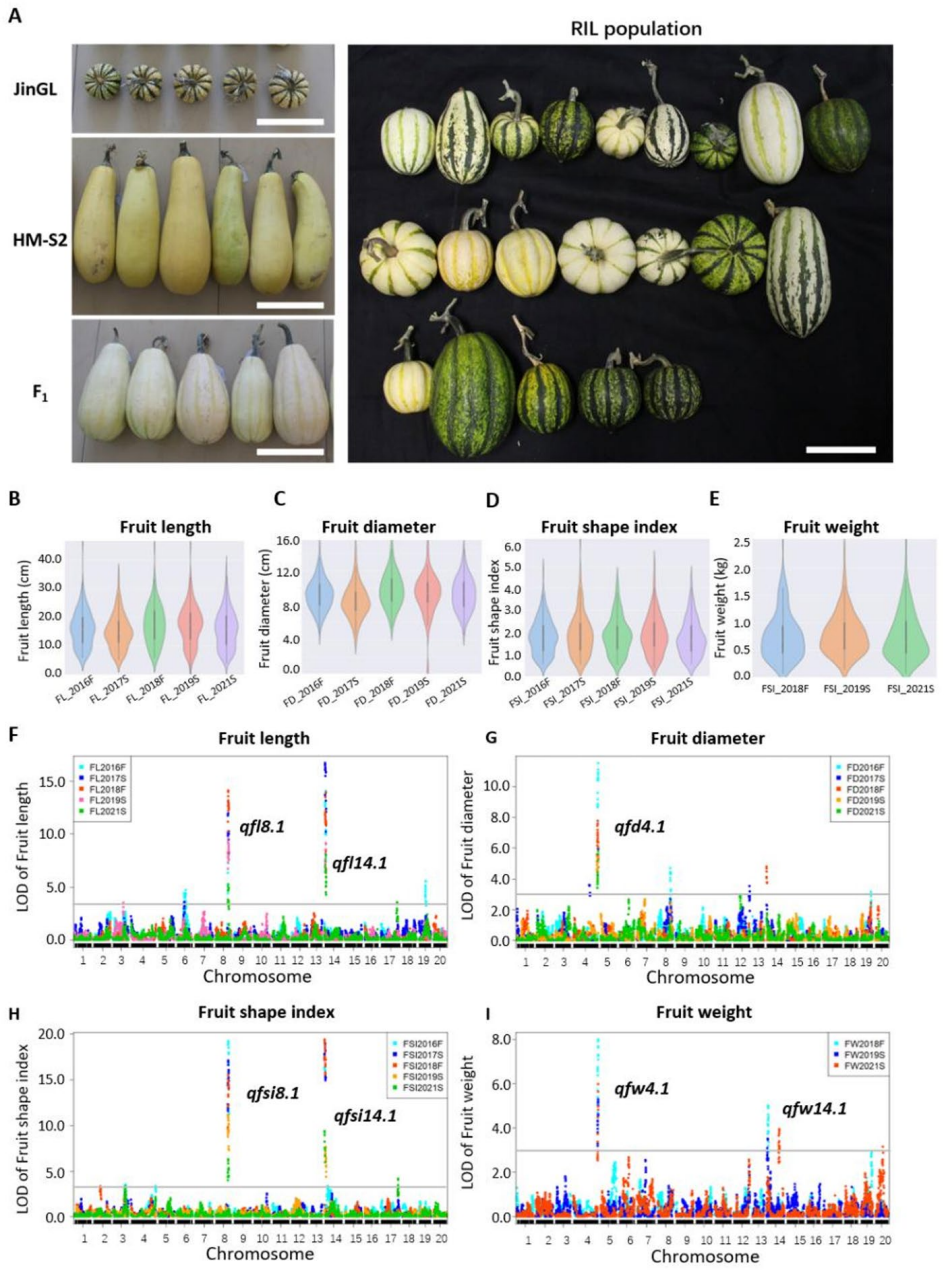
QTL analysis of fruit shape-related traits in the RIL population. **(A)** Fruit phenotypes of JinGL, HM-S2, F_1_, and representative RILs. Scale bar = 15 cm (**B-E**) Violin plots depicting phenotypic variation in FL, FD, FSI, and FW among the RIL population. (**F-I**) LOD profiles for FL (F), FD (G), FSI (H), and FW (I) from QTL analysis in different environments. One or two major-effect QTLs were detected for the respective trait. Two QTLs associated with fruit length were mapped to Chr08 and Chr14, designated *qfl8.1* and *qfl14.1*, respectively. Note that *qfd4.1* and *qfw4.1*, *qfl8.1* and *qfsi8.1*, *qfl14.1*, *qfsi14.1* and *qfw14.1* were colocalized in the same interval

Phenotypic data for fruit size/shape traits (FL, FD, FW, FSI) were collected in multiple experiments (Table 1). FL, FD, and FW were significantly smaller in JinGL than in HM-S2, while F_1_ progeny exhibited intermediate values (Fig. 3A; Table 3). Pearson’s correlation analysis revealed that FL was not significantly correlated with FD but had a strong negative correlation with FSI (*r*=−0.80, *P* < 0.001) and a positive correlation with FW (*r* = 0.63, *P* < 0.001). FSI displayed a moderate negative correlation with FW (*r*=−0.28, *P* < 0.01) (Table S2). Violin plots of the four traits in the RIL population (Fig. 3B-E) revealed continuous distributions, consistent with their quantitative inheritance.

QTL analyses using multiple-year/multi-environment data (Table 4; Fig. 3F-I) identified seven QTLs across three chromosomes for the four traits: two FL QTLs (*qfl8.1* on Chr08/LG17 and *qfl14.1* on Chr14/LG03); two FSI QTLs, *qfsi8.1* and *qfsi14.1* on the same chromosomes as the FL QTLs; one FD QTL (*qfd4.1* on Chr04/LG01); and two FW QTLs (*qfw4.1* on Chr04/LG01 and *qfw14.1* on Chr14/LG03). Four of these QTLs (*qfl8.1*,* qfl14.1*, *qfd4.1* and *qfw4.1*) represent novel discoveries, not previously reported in *Cucurbita pepo*. For *qfd4.1*, LOD scores ranged from 5.1 to 12.3, explaining 14.9–21.9% of PVE. *qfw4.1* exhibited LOD scores of 5.2–8.1 and explained 14.5–19.6% of PVE across trials. Six of the seven QTLs, including *qfl8.1/qfsi8.1*, *qfl14.1/qfsi14.1*, and *qfd4.1/qfw4.1*, exhibited PVE > 10% and were detected in multiple environments, qualifying them as major-effect QTLs.

**Table 4:** QTL analysis for the RILs in different experiments (2016 spring to 2021 spring)

Traits	Environments	QTL detected	Chr	QTL Peak (cM)	Peak LOD Score	1.5 LOD interval		Phenotypic Variation (%)
						left (cM)	right (cM)	
Hypocotyl Length (HL)								
	2021 S-March	*hl3.1*	3	Marker87504 (123.311)	4.6	Marker85674 (117.883)	Marker88675 (128.406)	15.4
	2021 S-April	*hl3.1*	3	Marker85155 (115.797)	5.6	Marker82480 (110.057)	Marker87136 (121.039)	15.1
	2021 S-May	*hl3.1*	3	Marker90099 (140.072)	6.0	Marker89970 (137.232)	Marker91043 (143.456)	15.8
Plant Height (PH)								
	2016 S	*ph3.1*	3	Marker90287 (141.277)	28.4	Marker90099 (140.072)	Marker90350 (142.496)	52.2
	2018 S	*ph3.1*	3	c3.loc140 (140.000)	21.0	Marker89970 (137.232)	Marker90350 (142.496)	39.6
	2019 S	*ph3.1*	3	Marker90287 (141.277)	45.1	Marker90099 (140.072)	Marker90350 (142.496)	70.9
	2021 S	*ph3.1*	3	Marker90287 (141.277)	15.5	Marker90099 (140.072)	Marker90350 (142.496)	30.1
Fruit Length (FL)								
	2016 F	*fl8.1*	8	Marker238454 (119.872)	10.1	Marker238258 (118.674)	Marker240069 (123.476)	19.5
		*fl14.1*	14	Marker374452 (10.341)	14.1	Marker373382 (3.865)	Marker375003 (11.967)	23.7
	2017 S	*fl8.1*	8	Marker238906 (121.457)	14.0	Marker237955 (116.523)	Marker240069 (123.476)	23.1
		*fl14.1*	14	Marker373816 (5.007)	17.3	Marker373382 (3.865)	Marker374356 (8.673)	29.5
	2018 F	*fl8.1*	8	Marker238454 (119.872)	13.9	Marker238258 (118.674)	Marker240232 (124.984)	26.0
		*fl14.1*	14	Marker374562 (10.634)	12.3	Marker373358 (4.168)	Marker375003 (11.967)	26.7
	2019 S	*fl8.1*	8	Marker238906 (121.457)	9.6	Marker238258 (118.674)	Marker240232 (124.984)	20.9
		*fl14.1*	14	Marker374452 (10.341)	9.1	Marker374286 (7.741)	Marker375003 (11.967)	16.2
	2021 S	*fl8.1*	8	Marker238906 (121.457)	5.0	Marker237947 (116.202)	Marker240069 (123.476)	14.6
		*fl14.1*	14	Marker374562 (10.634)	7.9	Marker373358 (4.168)	Marker375028 (12.440)	17.9
Fruit Diameter (FD)								
	2016 F	*fd4.1*	4	Marker146063 (237.625)	12.3	Marker143915 (235.391)	Marker146063 (237.625)	21.9
	2017 S	*fd4.1*	4	Marker146064 (237.331)	7.4	Marker142519 (233.328)	Marker146063 (237.625)	17.8
	2018 F	*fd4.1*	4	Marker141456 (232.442)	7.8	Marker141128 (231.266)	Marker146461 (236.273)	17.2
	2019 S	*fd4.1*	4	Marker141116 (231.560)	5.1	Marker138657 (226.342)	Marker145792 (236.743)	14.9
	2021 S	*fd4.1*	4	Marker146063 (237.625)	5.4	Marker142777 (234.214)	Marker146063 (237.625)	21.7
Fruit Shape Index (FSI)								
	2016 F	*fsi8.1*	8	Marker238906 (121.457)	19.3	Marker238258 (118.674)	Marker240069 (123.476)	30.9
		*fsi14.1*	14	Marker372698 (0.234)	19.3	Marker372697 (0.000)	Marker372885 (1.528)	28.3
	2017 S	*fsi8.1*	8	Marker238906 (121.457)	16.4	Marker238093 (117.781)	Marker240069 (123.476)	28.0
		*fsi14.1*	14	Marker373223 (3.255)	18.1	Marker372697 (0.000)	Marker373888 (6.572)	29.2
	2018 F	*fsi8.1*	8	Marker238454 (119.872)	15.7	Marker238258 (118.674)	Marker240176 (124.385)	27.0
		*fsi14.1*	14	Marker372698 (0.234)	19.4	Marker372697 (0.000)	Marker373888 (6.572)	34.3
	2019 S	*fsi8.1*	8	Marker238906 (121.457)	10.8	Marker238258 (118.674)	Marker240069 (123.476)	23.9
		*fsi14.1*	14	Marker374562 (10.634)	7.3	Marker373358 (4.168)	Marker375187 (13.527)	15.9
	2021 S	*fsi8.1*	8	Marker238454 (119.872)	6.3	Marker238093 (117.781)	Marker240069 (123.476)	16.3
		*fsi14.1*	14	Marker372697 (0.000)	9.4	Marker372697 (0.000)	Marker372904 (1.645)	20.1
Fruit Weight (FW)								
	2018 F	*fw4.1*	4	Marker146461 (236.273)	8.1	Marker144063 (234.802)	Marker146063 (237.625)	17.7
		*fw14.1*	14	Marker375294 (14.136)	4.9	Marker374637 (9.287)	Marker376116 (18.367)	10.3
	2019 S	*fw4.1*	4	Marker146063 (237.625)	5.2	Marker142450 (233.920)	Marker146063 (237.625)	14.5
		*fw14.1*	14	Marker374562 (10.634)	3.5	Marker373358 (4.168)	Marker375480 (14.447)	6.9
	2021 S	*fw4.1*	4	Marker146737 (237.037)	5.9	Marker145922 (235.685)	Marker146063 (237.625)	19.6
		*fw14.1*	14	Marker400771 (119.833)	3.2	Marker373358 (4.168)	Marker376003 (16.861)	9.7

### Candidate gene analysis for fruit size/shape-related traits

The major-effect QTL *qfl8.1/qfsi8.1* was flanked by two SLAF markers (238258 and 240069). This 472-kb interval corresponds to positions 1 180 234 − 1 653 023 on LG17 of the *Cucurbita pepo* genome and positions 6 540 761 − 6 828 332 on Chr08 of the *Cucurbita maxima* genome. Within this region, approximately 68 genes were predicted (Table S3). Sequence variation analysis between the parental lines (JinGL and HM-S2) identified 179 InDels in the exons of 47 genes (Table S4). Among them, 41 InDels were located in the 5’-UTR regions of 22 genes, and 37 InDels were in the coding region sequences (CDS) of 17 genes (Table S5).

Among the candidate genes, *Cp4.1LG17g02030* encodes Indole-3-acetic acid inducible 12 (IAA12; known as an auxin-responsive protein), a component of the auxin signaling pathway. *Cp4.1LG17g02010* encodes a Calcium-dependent lipid-binding (CaLB) domain protein, potentially involved in mediating calcium influx and activating signaling pathways such as those regulated by abscisic acid (ABA). *Cp4.1LG17g02260* encodes an Asn/Gln amidotransferase subunit B, while *Cp4.1LG17g02000* (an extensin protein-rich gene) is associated with cell elongation. *Cp4.1LG17g02240*, encoding a Zinc finger CONSTANS-like 4 protein, is associated with flower formation. Two genes, *Cp4.1LG17g02340* (embryo sac development arrest 6) and *Cp4.1LG17g02130* (encoding a peptide transporter) exist as tandem repeats in the *Cucurbita pepo* genome but as a single copy present in *Cucurbita maxima*.

**Fig. 4:**
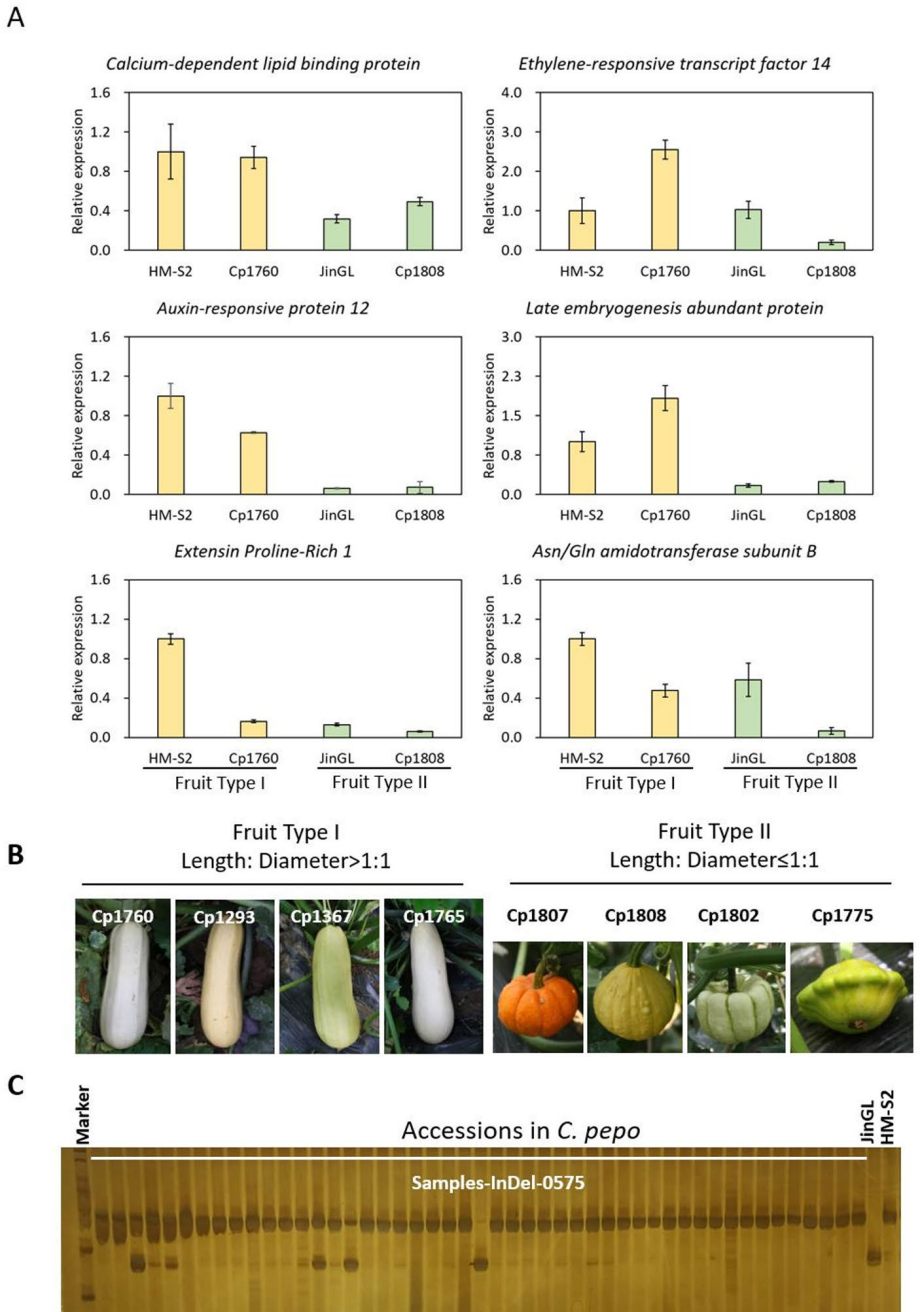
Relative expression of six candidate genes and validation of a molecular marker in *Cucurbita pepo* accessions. (**A**) Relative expression of six candidate genes in fruit, including *Calcium-dependent lipid binding protein*, *Auxin-responsive protein 12*, *Extensin Prolin-rich 1*, *Ethylene-responsive transcript factor 14*, *Late embryogenesis abundant protein*, and *Asn/Gln amido transferase subunit B*. (**B**) Typical fruit with FSI > 1.0 (Type I) and FSI ≤ 1.0 (Type II among *Cucurbita pepo* accessions. (**C**) Gel image showing marker patterns of InDel-0575 in a subset of 224 *Cucurbita pepo* accessions

Six candidate genes were selected for expression analysis via qRT-PCR in two *Cucurbita pepo* fruit groups: elongated fruits (Type I; FSI > 1.0) and round fruits (Type II; FSI ≤ 1.0) (Fig. 4A-B). Expression levels of *Cp4.1LG17g02010* (*Calcium-dependent lipid binding protein*), *Cp4.1LG17g02030* (*IAA12*), and *Cp4.1LG17g02160* (*Late embryogenesis abundant protein*) correlated with fruit shape: higher expression in elongated-fruited accessions, and lower expression in round-fruited accessions. These results suggest their potential roles in the regulation of fruit morphology.

### Development and validation of molecular markers associated with *qfl8.1/qfsi8.1*

An InDel marker, InDel-0575, was developed in the vicinity *Cp4.1LG17g02030* within the *qfl8.1/qfsi8.1* QTL interval. This marker was used to evaluate the association between its alleles and fruit shape in 224 *Cucurbita pepo* germplasm accessions (Fig. 4C). These accessions exhibited diverse fruit shapes, ranging from round (length-to-diameter ratio ≤ 1:1) to elongated (length-to-diameter > 1:1). Genotypic and phenotypic data for these accessions are summearized in Table S6. The InDel marker showed 98.0% accuracy in elongated-fruited accessions (194/198) and 96.1% accuracy in round-fruited accessions (25/26), confirming the high reliability and effectiveness of InDel-0575 as a trait-linked marker for fruit shape.

## Discussion

### Diverse RILs as effective tools for genetic mapping of horticultural traits in *Cucurbita pepo*

Following the first report by Montero-Pau et al. (2017), this study reports the second recombinant inbred line (RIL) population developed for genetic mapping in *Cucurbita pepo*. The parental line ‘JinGL’ carries more favorable traits, making it better suited for breeding programs in China and East Asia. The high-density genetic map offers several advantages: First, compared to previous low-density linkage maps or small F₂ populations, it has increased the marker density and the size of the RIL population. This improved the efficiency of detecting QTLs, especially for traits controlled by multiple genes with small effects. Second, the map contains 20 linkage groups, matching the chromosome number and order within *Cucurbita pepo* (*n* = 20), ensuring a precise correspondence with the karyotype. Third, these genetic markers show a strong linear alignment with their physical positions in the reference genomes of *Cucurbita maxima* and *Cucurbita moschata* (Zhong et al. 2017; Sun et al. 2017; Zeng et al. 2024), providing reliable genomic coordinates for future map-based cloning and marker-assisted breeding.

For instance, regarding plant height in *Cucurbita pepo*, while Montero-Pau et al. (2017) reported substantial phenotypic differences in vine length and node number between parental lines of a zucchini × scallop RIL population, no statistically significant QTL governing these traits were identified in their RIL population. In contrast, our study detected QTLs with up to 50% PVE for plant height and developed molecular markers tightly linked to these loci. This divergent result highlights the potential value of our genetic map and RIL population for identifying novel QTLs.

In addition to plant height, we also investigated hypocotyl length, a trait closely associated with seedling uniformity. To our knowledge, no prior QTL mapping of this trait has been reported in *Cucurbita pepo*. Here, we identified a major-effect QTL related to both hypocotyl length (*qhl3.1*) and adult plant height (*qph3.1*), which is located in the same genomic region (Fig. 2; Table 3). Subsequently, fine-mapping of hypocotyl length using additional genetic populations will aim to determine whether these two traits are regulated by the same genetic locus as those underlying plant height in zucchini.

### Novel QTLs for fruit size/shape-related traits in *Cucurbita pepo*

Prior QTL mapping studies in *Cucurbita pepo* have focused on fruit size and shape traits (Esteras et al. 2012; Montero-Pau et al. 2017). Esteras et al. (2012) identified QTLs for fruit length (FL), diameter (FD), and shape index (FSI) using a zucchini × scallop cross. Montero-Pau et al. (2017) later constructed a 21-linkage-group genetic map and mapped fruit morphology QTLs thereon, identifying one major QTL (MFSh_3 on LG03) and two minor QTLs (MFSh_4 on LG04, MFSh_5 on LG05) associated with mature fruit shape. Due to the availability of *Cucurbita pepo* v3.2 sequence data, we could only tentatively compare QTL physical positions with those from previous studies. This limitation prompted us to resequence the parental genomes and construct a high-density genetic map in the present study.

In our current investigation, two major-effect QTLs for FSI, designated as *qfsi8.1* and *qfsi14.1*, were detected on Chr08/LG17 and Chr14/LG03, with the highest LOD scores (19.3–19.4) and the PVE ranging from 30.9 to 34.3%. Notably, the genomic region harboring MFSh_3 (specifically, CP32_scaffold00038_1385297 − CP32_scaffold00038_1853138) appears to colocalize with *qfsi14.1*, which is delimited by the flanking markers Marker373382 and Marker375003 on Chr14/LG03 in our study (Table 4). No fruit shape-related loci were reported on Chr08/LG17 in prior studies. Thus, our findings represent the first identification of *qfsi8.1*, delimited by Marker238258 and Marker240069 on Chr08/LG17, as a novel major-effect QTL controlling FSI in *Cucurbita pepo*.

Our study also reports novel QTLs associated with fruit diameter (FD) and fruit weight (FW). QTLs for FW have not been reported in previous studies, which is likely due to the small phenotypic differences in fruit weight between the parental lines used in those studies (Montero-Pau et al. 2017). In this study, we identified a novel FD QTL (designated *qfd4.1*) on Chr04/LG01 (20 265 755 − 20 818 840) within its 1.5-LOD support interval, and LOD peaks ranged from 5.1 to 12.3. Additionally, two novel FW QTLs (*qfw4.1* and *qfw14.1*) were identified on Chr04/LG01 (20 319 126 − 20 818 840) and Chr14/LG03 (743 413 − 1 235 968), respectively.

Significant colocalization of fruit-related QTLs was detected on Chr04, Chr08, and Chr14, suggesting that these chromosomal regions are strongly associated with phenotypic variation in fruit traits. These loci may contribute to fruit morphology to varying extents, with their corresponding candidate genes potentially involved in genetic interactions. Notably, QTLs *MFLe_3*, *MFSh_3* (reported in previous studies), and those we identified *qfl14.1*, *qfsi14.1*, and *qfw14.1* colocalized within the same genomic interval of Chr14/LG03. This finding highlights the complex genetic interactions underlying multiple fruit traits in this region and implies conserved mechanisms regulating fruit morphogenesis in different cultivar groups in *Cucurbita pepo*.

Interestingly, a prior study in *Cucurbita maxima* also reported several QTLs associated with fruit shape, which exhibited colocalization with QTLs detected in our study in *Cucurbita pepo*. These included QTLs for fruit length, diameter, and shape index, as reported by Kaźmińska et al. (2020). Notably, *qfw14.1* identified in *Cucurbita maxima* was found to colocalize with our *qfl14.1/qfsi14.1* in *Cucurbita pepo* on Chr14. Additionally, another QTL (*qfw4.1*) showed colocalization with our FD QTL (*qfd4.1*) on Chr04/LG01. While the candidate genes underlying these QTLs in both *Cucurbita maxima* and *Cucurbita pepo* remain to be identified, this observation highlights the potential for shared genetic determinants that influence fruit shape traits during selection and domestication (Monforte et al. 2014; Kaźmińska et al. 2020).

### Prediction and identification of candidate genes for fruit length-related QTL *qfl8.1*

In this study, we detected two major QTLs for fruit length: *qfl8.1* and *qfl14.1*. In model plants such as *Arabidopsis*, *Cucumis sativus*, and *Solanum lycopersicum*, extensive studies have explored QTL mapping and functional analysis of candidate genes involved in fruit shape determination. Key determinants identified include genes *SUN*, *OVATE*, *FASCIATED* (*FAS*), *WUSCHEL*, *CLAVATA*, *MADS*-domain transcription factors, and phytohormone signaling components (Dou et al. 2018; Zhao et al. 2019; Pan et al. 2017; 2020). For *qfl14.1*, we delimited a 234-kb physical interval (626 501 – 861 016 on Chr14/LG03) containing 51 predicted genes. None of the previously reported fruit shape-related genes mapped to this interval, indicating the discovery of a potentially novel QTL governing fruit length.

The second QTL, *qfl8.1* on Chr08/LG17, was narrowed to a 472-kb region harboring 68 predicted genes. To our knowledge, no fruit-shape-associated QTLs or annotated candidate genes have been reported on Chr08/LG17 in *Cucurbita maxima* or *Cucurbita moschata*. This finding highlights *qfl8.1* as a unique regulatory locus for fruit morphology in *Cucurbita pepo*, potentially representing a unique genetic mechanism underlying fruit shape variation.

Through sequence polymorphism analysis, qRT-PCR analysis, and molecular marker validation in germplasm accessions, we were able to narrow down the candidate gene list for this region. Among them, *Cp4.1LG17g02030*, which encodes an auxin-responsive protein, exhibits 69.7% sequence identity with *Arabidopsis* indole-3-acetic acid-inducible 12 (IAA12; also known as BODENLOS). In *Cucumis sativus*, the regulation of auxin homeostasis is crucial for determining fruit length (Zhao et al. 2019). In *Arabidopsis thaliana*, IAA12 forms a complex with SCF^TIR1^ and ARF5/MOP, mediating auxin signaling and regulating fruit morphogenesis (Gray et al. 2001; Jia et al. 2021; Chen et al. 2025). The fruit shape-related molecular marker InDel-0575, developed in this study, was found to be tightly linked to *Cp4.1LG17g02030*. Validation across long- and round-fruited germplasm accessions showed 96.1–98.0% consistency, indicating that this gene (*Cp4.1LG17g02030/CpIAA12*) or other genes in this genomic region likely contribute to the regulation of fruit-related traits.

Additionally, *Cp4.1LG17g02010*, which encodes a calcium-dependent lipid-binding family protein, was previously identified as a candidate gene for fruit length in *Cucurbita pepo* by Montero-Pau et al. (2017) and Xanthopoulou et al. (2019), though its functional role in fruit development remains to be characterized. Fine-mapping and functional characterization of candidate genes involved in fruit length regulation are currently underway in *Cucurbita pepo*. In future studies, it will be critical to validate the functions of the candidate gene identified here through a combination of transgenic complementation assays and CRISPR/*Cas9* editing technology. Meanwhile, *in situ* hybridization will be used to analyze the expression patterns of the candidate gene, while yeast two-hybrid assays will be used to identify the interacting proteins. These approaches will help elucidate the potential mechanisms underlying fruit shape regulation in *Cucurbita pepo*. The role of phytohormone signaling in these regulatory pathways warrants further investigation. These functional validation efforts will help establish a direct link between the candidate genes and the observed phenotypic variations, providing a more robust theoretical basis for their application in zucchini breeding.

## Supplementary Information

Below is the link to the electronic supplementary material.

Supplementary Material 1 (XLXS 50.7 KB)

Supplementary Material 2 (PDF 264 KB)

## Data Availability

The data presented in the current study are available in the article and Supplementary Materials from the corresponding author upon request.
