# Supplementary material for: Development of recombinant inbred lines and QTL analysis of plant height and fruit shape-related traits in Cucurbita pepo L
Source: Mol Breed. 2025 Sep 9;45(9):74. doi: 10.1007/s11032-025-01592-y (PMC12420555; doi:10.1007/s11032-025-01592-y)
Supplement: Supplementary file 2 — Supplementary Material 2 (PDF 264 KB) [file 11032_2025_1592_MOESM2_ESM.pdf]

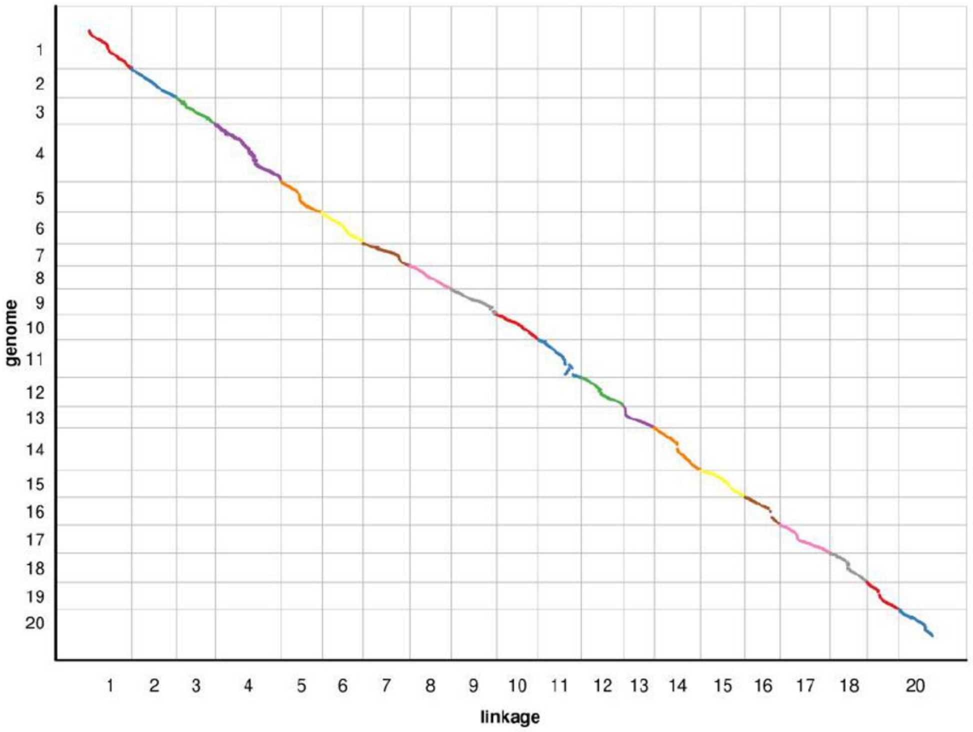

Fig. S1 Alignment of the high-density genetic map from the present study with the 20 chromosomes of the *Cucurbita maxima* cv. Rimu genome assembly.

The abscissa represents genetic distance (cM) in each linkage group. The ordinate represented physical length (Mb) of each Chromosome. Colors indicate the degree of collinearity between linkage groups and their corresponding chromosomes.
